# The Two Cultures

**DOI:** 10.15694/mep.2018.0000174.1

**Published:** 2018-08-21

**Authors:** Mohammad Razai

**Affiliations:** 1 St George's University of London

**Keywords:** Psychological safety, postgraduate training, Professional regulation, patient safety, understaffing

## Abstract

This article was migrated. The article was marked as recommended.

Doctors are not only under unprecedented level of scrutiny and regulation, the professional standard and expectation of performance has never been higher. However, those who work in circumstances where the most crucial resources such as adequate personnel are lacking, find the reality far removed from the high-toned language of the professional code of practice. The clash of the real and the ideal inevitably lead to catastrophic consequences as illustrated by the recent high-profile cases. Systemic failures are overlooked and diminished but the actions of individual doctors are heavily probed. This has created an unsafe culture of blame and punishment where doctors are set up to fail within a struggle system. What are the consequences of this for the profession and how to start dealing with them?

## The Clash of the Real and the Ideal

Anyone who has worked in Britain’s Health Service cannot but see two distinct cultures developing, each moving in opposite directions. This widening chasm will not only rip apart medicine as a profession but will do irreparable damage to the health of the population. Something must be done. The two cultures stem from two realms of existence, as one is reaching for the heavens the other is burrowing deeper into earthly existence. On the one hand there are suited bodies with long titles residing in comfortable boardrooms, producing and propagating sublime professional standards. These adjective-laden documents describe the virtues that a doctor must possess and retain the outstanding care that ought to be provided. All the time. To err is not acceptable and so on.

They have borrowed the language and structure of medieval ecclesiastical institutions. The sanctimonious code of practice, the inquisition and ex-communication of deviants and the inevitable sense of guilt, shame and loss that such shunning brings to the outcast. Whilst the prevailing language of our society is of rights, in medicine it is of duty and responsibility. However, the culture of ideal is aimed at saints. Mere mortals who have to operate within the constraints of the human condition and their environment are condemned to work in the real world.

Imagine this. You get up at the crack of dawn – sleep-deprived because you left work late the previous day – and arrive at a busy hospital in an inner-city area. The department is severely understaffed, and you have previously raised concerns about various issues including staffing levels and patient safety. On the day, your task is to see 50 inpatients with a jobs list of at least 40 items including performing vital diagnostic tests, preparing discharge paperwork to taking blood samples and updating family members. As the ward round continues you are constantly interrupted by a deluge of bleeps every few seconds from A&E, wards, peripheral hospitals and General Practices.

The consultant and registrar have both gone to theatre. At least 7 patients are waiting for you in A&E. You make your way down to see the most unwell but waylaid by an angry relative who is not happy with the care their loved one has received and demands an explanation. You are paged several times whilst speaking with the upset next-of-kin. You answer the bleep and hear the voice of a stern ward clerk asking why a discharge summary has not been completed for a patient who left hospital a few days ago. It is against the hospital policy for patients to leave without proper paperwork and even worse that there should be such a long delay. You try to assuage her frustration by acknowledging its importance and reassure that it will be done but it is not a priority at the moment. You realise you have not seen, heard or looked after this patient before. She demands that it must be completed. To make her point, she has done an incident report for the delay and has put your name in the report as one of the culprits.

At this point the phone rings, it is your consultant with a gruff tone asking you why you are not answering your bleep. You apologise and explain that you were on the phone. You must see another patient referred by the A&E consultant urgently before she breaches the waiting time protocol of the department.  You traipse down to A&E, on the way realise you have not eaten, drunk or been to relieve yourself for about 10 hours. Eventually you sit down to complete the paperwork but your login details does not work and you are unable to contact the IT department. This is a snapshot of typical on-call in one of my recent hospital jobs.

When the Bawa-Garba tragedy came to light many of us recoiled in fear because it was too close to the bone. Here was an unsupported junior doctor on her first day back to work, in a severely understaffed department with multiple serious systemic failures, was set up to fail and received an exemplary punishment for failing. She was judged as if she was working under perfect conditions endowed with superhuman qualities. They threw the book at her and measured her performance against the culture of cosy boardrooms.

The clash of the real and ideal inevitably create a tsunami of emotional and physical turmoil with a profound sense of alienation, inadequacy and failure. The steep fall from grace ismarried with fear and loss – fear of making a career-ending mistake, being hauled before the GMC and blamed for the errors. The loss of face, professional standing and a livelihood.

## Take Home Messages

• The vast majority of doctors are honest, compassionate, competent people. The haranguing of individuals by an organisation that has become a leviathan is the road to nowhere.
• Regulation should be a local issue at the place of work where doctors already have their appraisal and revalidation. Any complaints and concerns about conduct within this framework could be swiftly and efficiently addressed.
• The GMC’s role should be to ensure the safety and adequacy of the system such as the quality of undergraduate and postgraduate training and education, hospital staffing levels and the appropriate funding of the health service.

## Notes On Contributors

Dr Mohammad Razai BSc MB BChir MA, FRSA is clinical academic fellow at St George’s University of London.
