# Supplementary material for: The Two Cultures
Source: MedEdPublish (2016). 2018 Aug 21;7:174. [Version 1] doi: 10.15694/mep.2018.0000174.1 (PMC10701840; doi:10.15694/mep.2018.0000174.1)
Supplement: Peer review history

# Reviewer report: Reviewer response for version 1

**DOI:** 10.21956/mep.19641.r29268

**Published:** 2018-09-11

**Authors:** David Taylor

**Affiliations:** 1 Gulf Medical University, Ajman, UAE

This review has been migrated. The reviewer awarded 4 stars out of 5 The author of this strongly written piece clearly finds themselves at the interface between the ideal and the real. Recently, elsewhere in this journal, we have been reading and thinking about the patient narrative, and there is a tendency to gloss over the doctor’s narrative. I have colleagues overseas who work under appalling circumstances, their reality is even worse, but is not overlaid with the ideal that is expected in the more developed world.The author has made his personal opinions clear. I hope, though, that someone with the skills and time will find a way of studying the narratives of junior doctors caught between the real and the ideal.

Reviewer Expertise:

NA

No decision status is available

# Reviewer report: Reviewer response for version 1

**DOI:** 10.21956/mep.19641.r29266

**Published:** 2018-09-04

**Authors:** P Ravi Shankar

**Affiliations:** 1 American International Medical University

This review has been migrated. The reviewer awarded 4 stars out of 5 This is a passionately written piece by a doctor which draws into focus the stark dichotomy between ‘idealistic’ standards set in boardrooms and the hard reality of clinical practice. The author describes very well a typical on-call day of a junior doctor. The situation described is from the United Kingdom but a similar situation exists in many developing nations. The issue of violence against doctors is a serious one in many nations and doctors have lost their lives at the hands of angry relatives and mob elements. Doctors are often the scapegoat for systemic failures and other shortcomings. The author writes very well and there are issues here which have to be addressed by society, health policy makers, administrators and others to ensure a good quality of medical care.

Reviewer Expertise:

NA

No decision status is available

# Reviewer report: Reviewer response for version 1

**DOI:** 10.21956/mep.19641.r29267

**Published:** 2018-08-28

**Authors:** Johnny Lyon Maris

**Affiliations:** 1 Southampton GP Education Unit

This review has been migrated. The reviewer awarded 3 stars out of 5 As a forum for personal opinion pieces, MedEdPublish certainly can perform this function alongside education research. The author has passion and writes about a personal experience which leaves the reader cold. As a reviewer I would like to see potential solutions, which have been hinted at in the take home messages, formulated into the body of the opinion piece. This needs to understand the complexity and politics of a healthcare system and be applicable to the broad audience of MedEdPublish not just the UK.The future of all health systems is in the hands of the training workforce, who will be the service providers in the near future. Systems can only change by being informed and guided by specialists in training, who will provide the healthcare workforce for the next 35 years. Reference must be made to experienced clinicians, but policy makers realise this group will retire over the next 10 years or so.

Reviewer Expertise:

NA

No decision status is available

# Reviewer report: Reviewer response for version 1

**DOI:** 10.21956/mep.19641.r29265

**Published:** 2018-08-21

**Authors:** Trevor Gibbs

**Affiliations:** 1 AMEE

This review has been migrated. The reviewer awarded 4 stars out of 5 A very emotive personal view that draws attention to the UK system, although I am very sure that similar and different pressures abound when one looks around the world and I suppose that some global partners would see that the UK has it easy when compared to their situation within a war-torn country, or a country in political upheaval etc. This should not however detract us from listening to all sides of the story.It is very difficult to rate such an opinion piece and the rating I give it here does not reflect my personal views, I think they do not matter in this case. I do base my rating on the way the author has expressed himself and how the paper demonstrates the passion and frustration that can be felt as one reads.

Reviewer Expertise:

NA

No decision status is available
